# The Reverse Transcription Inhibitor Abacavir Shows Anticancer Activity in Prostate Cancer Cell Lines

**DOI:** 10.1371/journal.pone.0014221

**Published:** 2010-12-03

**Authors:** Francesca Carlini, Barbara Ridolfi, Agnese Molinari, Chiara Parisi, Giuseppina Bozzuto, Laura Toccacieli, Giuseppe Formisano, Daniela De Orsi, Silvia Paradisi, OlÌ Maria Victoria Grober, Maria Ravo, Alessandro Weisz, Romano Arcieri, Stefano Vella, Simona Gaudi

**Affiliations:** 1 Department of Therapeutic Research and Drug Evaluation, Istituto Superiore di Sanità, Rome, Italy; 2 Department of Technology and Health, Istituto Superiore di Sanità, Rome, Italy; 3 Department of Cell Biology and Neurosciences, Istituto Superiore di Sanità, Rome, Italy; 4 Department of General Pathology, Second University of Naples, Naples, Italy; 5 Laboratory of Molecular Medicine, Faculty of Medicine, University of Salerno, Salerno, Italy; Baylor College of Medicine, United States of America

## Abstract

**Background:**

Transposable Elements (TEs) comprise nearly 45% of the entire genome and are part of sophisticated regulatory network systems that control developmental processes in normal and pathological conditions. The retroviral/retrotransposon gene machinery consists mainly of Long Interspersed Nuclear Elements (LINEs-1) and Human Endogenous Retroviruses (HERVs) that code for their own endogenous reverse transcriptase (RT). Interestingly, RT is typically expressed at high levels in cancer cells. Recent studies report that RT inhibition by non-nucleoside reverse transcriptase inhibitors (NNRTIs) induces growth arrest and cell differentiation *in vitro* and antagonizes growth of human tumors in animal model. In the present study we analyze the anticancer activity of Abacavir (ABC), a nucleoside reverse transcription inhibitor (NRTI), on PC3 and LNCaP prostate cancer cell lines.

**Principal Findings:**

ABC significantly reduces cell growth, migration and invasion processes, considerably slows S phase progression, induces senescence and cell death in prostate cancer cells. Consistent with these observations, microarray analysis on PC3 cells shows that ABC induces specific and dose-dependent changes in gene expression, involving multiple cellular pathways. Notably, by quantitative Real-Time PCR we found that LINE-1 ORF1 and ORF2 mRNA levels were significantly up-regulated by ABC treatment.

**Conclusions:**

Our results demonstrate the potential of ABC as anticancer agent able to induce antiproliferative activity and trigger senescence in prostate cancer cells. Noteworthy, we show that ABC elicits up-regulation of LINE-1 expression, suggesting the involvement of these elements in the observed cellular modifications.

## Introduction

Cancer is a complex disease and its extreme phenotype variability can not be exhaustively described and explained solely by the gene-environment interactions. Unexpectedly, the completion of the human genome reveals that the true complexity of the genome has little to do with the number and heterogeneity of its genes.

Only 2% of the human genome codes for proteins, while the 45% consists of Transposable Elements (TEs) [1]. The TEs, initially thought to be mere intracellular parasites, were termed selfish or “junk” DNA, until further research revealed how this huge amount of non-protein-coding sequences scales up with organisms complexity, playing a critical role as a part of regulatory toolkit of the genome, by altering gene expression and driving genome evolution as well as the development of an organism [2]–[10]. TEs can be separated into two major classes: DNA-transposons (2.8%) and retrotransposons (42.2%). DNA-transposons amplify without an RNA intermediate, while retrotransposons rely on an RNA transcript that self-replicates with the aid of a reverse transcriptase (RT).

RTs encoded by Long Interspersed Nuclear Element-1 (LINE-1) and Human Endogenous Retroviruses (HERVs) were found to be associated with pathological and physiological processes. In particular, high expression levels of RT were found in germ cells, embryos, undifferentiated and transformed cells, while in differentiated non-pathological tissues they were barely expressed, suggesting a direct correlation with the proliferative potential of the cell [11]–[17].

Previous studies have demonstrated that a class of non-nucleoside RT inhibitors (NNRTIs), widely used in AIDS therapy, inhibits the endogenous RT activity in a number of murine and human cancer cell lines, reducing the cell growth rate and inducing differentiation [18]–[20]. Furthermore, the same biological effects were reproduced in RNA interfering experiments with specific *si*RNA directed against LINE-1 and HERV-K. These results clearly indicate that both classes of retroelements are linked in a functional network, involved in cell growth and tumorigenesis, but with distinct hierarchical roles, being LINE-1 able to control HERV-K expression [21]. These studies suggest that endogenous non-telomeric RT may represent a novel target in the development of therapeutic anticancer agents.

NNRTIs bind to a hydrophobic pocket, near to the RT active site. The binding induces a conformational change in the protein, affecting its affinity for the substrate and, consequently, inhibiting RT enzymatic activity. NNRTIs are classified as allosteric non-competitive RT inhibitors.

Another class of antiretroviral inhibitors that target RT is represented by the nucleoside reverse transcription inhibitors (NRTIs). Compared to the NNRTIs, they have a different mechanism of action. The NRTIs are nucleotide analogues that inhibit reverse transcription by being incorporated into the newly synthesized viral DNA (cDNA) and preventing its further elongation. They are classified as non-allosteric competitive substrate inhibitors. Among these, Abacavir (ABC) is successfully used in combination with other antiviral drugs in the treatment of HIV infection. It is converted by cellular enzymes to the active carbovir triphosphorilated form, an analogue of dGTP. With respect to other NRTIs, ABC shows a very low affinity for cellular DNA polymerases [22]. Additionally, Jones et al. [23] demonstrated with an *in vitro* LINE-1 retrotransposition assay that NRTIs have the ability to suppress LINE-1 retrotransposition, indicating the susceptibility of LINE-1 RT to this class of drugs.

Based on these evidences, we have investigated the antitumor activity of ABC on the hormone-dependent LNCaP and hormone-refractory PC3 prostate cancer cell lines. These metastatic cells are generally assumed to represent early and late stages of prostate cancer respectively. Until now, prostate cancer represents the most common noncutaneous cancer in man in the United States [24]. The mainstay treatment is the androgen ablation, but after an initial good response the disease progresses to hormone-refractory prostate cancer [25]. New therapy modalities are needed to prevent or treat this more lethal form of prostate cancer.

Here we demonstrate that ABC induces a significant decrease in cell growth rate and a delay of the cell cycle in S phase. A high percentage of senescent cells were observed and many of them were committed to death. Few hours of ABC exposure significantly reduced the potential of migration and invasion in prostate cancer cells. Moreover, a genome-wide expression analysis on PC3 cells revealed a dose-dependent gene regulation. Notably, ABC was able to modulate LINE-1 expression in both cell lines.

## Materials and Methods

### Cell Cultures and Treatments

The human prostate cancer cell lines PC3 (ATCC CRL-1435) and LNCaP (ATCC CRL-1740) and the non-transformed human fibroblast cell line WI-38 (ATCC CCL-75) were cultured according to the ATCC recommendations. Abacavir was purified from commercially available Ziagen tablets (GlaxoSmithKline, Research Triangle Park, NC 27709) by methanol extraction and purification grade assessed by HPLC. For drug experiments, ABC was dissolved in FBS-free medium and used at the concentration of 15 or 150 µM. Drug-containing fresh medium was changed every 48 h. For synchronization experiments cells were treated with 2 µg/ml aphidicolin for 18 h, then washed twice with PBS and released in fresh medium, containing or not ABC.

### RT Activity Assay

RT activity assays were performed by a minor modification of the method described by Mangiacasale et.al [18]. Cells (5×10^5^–10^6^) were lysed in 40 µl ice-cold lysis buffer (10 mM Tris-HCl pH 7.5, 1 mM MgCl_2_, 1 mM EGTA, 0.1 mM PMSF, 5 mM β-mercaptoethanol, 0.5% CHAPS, 10% glycerol). After three freeze-and-thaw cycles, cells were incubated for 30 min on ice and centrifuged for 30 min at 14 000 r.p.m. at 4°C. RT activity was tested using a ThermoScript RT–PCR system (Invitrogen) in 20 µl reactions containing 10 ng of MS2 phage RNA (Roche Diagnostics) and 30 pmol of MS2 reverse primer (see below) and substituting commercial RT with cell-free extract (24 µg total protein). Reaction mixtures were incubated at 55°C for 1 h followed by 5 min at 85°C. A 1 µl volume of *Escherichia coli* RNaseH (2 U/µl) was added to each sample and further incubated at 37°C for 20 min. Control reactions were set up by omitting cell extract (negative control), or adding 1 µl of ThermoScript RT (Invitrogen) (15 U/µl, positive control). A 2 µl volume from each reaction was mixed with 30 pmol each of forward (5′-TCCTGCTCAACTTCCTGTCGAG-3′) and reverse (5′-CACAGGTCAAACCTCCTAGGAATG-3′) MS2 primers and PCR-amplified using the Platinum PCR SuperMix (Invitrogen). PCR conditions were as follows: 94°C for 2 min, followed by 30 cycles of 94°C for 30 s, 58°C for 30 s and 72°C for 30 s. The amplification product is a 112-bp DNA fragment spanning positions 21–132 at the 5′ end of the MS2 RNA (GenBank V00642). PCR products were fractionated through 1.5% agarose gel electrophoresis.

### Cell Proliferation Assay

PC3, LNCaP and WI-38 cells were seeded at a density of 20,000 per well in 12-well plates, cultured for 24 h and then treated with ABC at the concentration of 15 or 150 µM. At 0, 24, 48, 72 and 96 h the number of trypan blue-excluding cells were counted in a Burker chamber. The experiments were performed three times in triplicate.

### Cell Cycle Analysis

Treated and untreated cells were harvested by trypsinization and washed with ice-cold PBS. DNA staining was performed by using the Cycle TEST™ PLUS DNA Reagent Kit (Becton & Dickinson). Cells were then analysed on a FACScan flow cytometer (Becton & Dickinson).

### Senescence Determination

Percentage of senescent cells was measured by detecting β-galactosidase activity at pH 6 with the Calbiochem Senescence Detection Kit. Data were quantified from more than 500 cell counts in three independent experiments.

### Fluorescence Microscopy

For morphometric analysis of the nuclei, treated and untreated cells were fixed with 3% paraformaldehyde and incubated with Hoechst 33258 solution (1 µg/ml) for 30 min at 37°C. For the staining of nucleoli, cells grown on 12 mm glass coverslips were fixed and permeabilized with methanol at −20°C for 10 min, incubated with antinuclear serum from an autoimmune patient (kindly provided by Dr. Maria Antonietta Amendolea) and then incubated with a fluorescein-linked goat anti-human IgG (Bio-Rad). The samples were analyzed by a CCD camera Nikon equipped Optiphot microscope (Tokyo, Japan). The morphometric analysis was performed with the Image J 1.37 software (Wayne Rasband, NIH, USA). For the statistical analysis Student's t -test was applied.

### Scanning Electron Microscopy (SEM)

Treated and untreated PC3 cells were fixed with 2% glutaraldehyde in 0.1 M cacodylate buffer pH 7.4 at room temperature for 30 min, post-fixed with 1% OsO_4_ in the same buffer, dehydrated through a graded ethanol series, critical point dried with CO_2_ and gold coated by sputtering. Samples were examined with a Cambridge Stereoscan 360 scanning electron microscope (Cambridge Instruments Ltd, Cambridge, UK).

### Cell migration and invasion assays

Assays were performed by a modification of the method described by Albini and colleagues [26]. For migration and invasion assays, filters 8.0 µm pore (Falcon) were used. Cells, harvested and suspended in RPMI at a concentration of 1×10^6^ cells/ml, were added to each filter and 3 ml of RPMI containing 10% FBS were added to the well underneath the insert. For invasion assay filters were coated with Matrigel™ (Sigma) diluted to 1 mg/ml in serum-free RPMI medium. Cells were incubated at 37°C for 18 h. Afterwards, the inner side of the filter was wiped with a wet swab to remove the cells while the outer side of the insert was rinsed with PBS and stained with 0.25% crystal violet (Sigma) for 10 min. The filters were then viewed under a CCD camera Nikon equipped Optiphot microscope (Tokyo, Japan) and the percent of area occupied by migrated or invading cells was analyzed by Optilab software (Graftek Mirmande, France).

### Microarray Analysis

Total RNA was isolated using RNase Kit (Qiagen). For each sample, 500 ng of RNA were synthesized to biotinylated cRNA using Illumina RNA Amplification Kit (Ambion, Inc., Austin, TX). RNA and cDNA concentration was determined with a Nanodrop spectrophotometer (NanoDrop, Wilmington, Delaware, USA) and its quality was assessed with an Agilent 2100 Bioanalyzer (Agilent Technologies, Milano, Italy). From each sample, technical replicates were produced and 750 ng cRNA were hybridized for 18 h to HumanRef-8 v2 Expression BeadChips (Illumina Inc., San Diego, CA, USA) according to the manufacturer's protocol. Intensity files were loaded into the Illumina BeadStudio 3.0.19.0 software and normalized with the average algorithm. Technical replicates of each sample were grouped together and genes with a detection p-value <0.05 were considered as detected. Genes with Diff Score of ±40 (p-value of 0.0001) were considered as differentially expressed genes. Microarray data are MIAME compliant and the raw data have been deposited in the ArrayExpress database (http://www.ebi.ac.uk/microarray-as/ae), with Accession number: E-TABM-532.

Ingenuity Pathway Analysis 7 (Ingenuity Systems®, www.ingenuity.com) was used to analyze the biological relationship of differentially expressed genes. The biological functions were evaluated according to the directions of the software. Fisher's exact test was used to calculate a p-value determining the probability that each biological function assigned to that data set is due to chance alone. A GO (Gene Ontology) enrichment analysis was also performed using DAVID software [27], [28].

### RT Assay and Quantification of LINE-1 mRNA Expression

Total RNA was isolated from cells untreated and treated with 15 and 150 µM ABC from 24, to 120 h. RNA was treated with TURBO DNase (Ambion) to remove contaminating genomic DNA. cDNAs were synthesized by the TaqMan High Capacity cDNA Reverse Transcription Kit (PE Applied Biosystems). Relative quantification PCR reactions were performed with TaqMan chemistry. LINE-1 transcripts were detected using custom primers and FAM-MGB-probes for LINE-1 ORF1 and ORF2 (PE Applied Biosystems), designed using the Primers Express software (Table S1). The LINE-1 sequence database used in this work is the L1base (http://l1base.molgen.mpg.de) [29]. The number of LINE-1 sequences targeted by probes are reported in Table S1. Each reaction was performed in a final volume of 25 µL containing 1 µL cDNA and 12.5 µL of TaqMan® Universal PCR Master Mix (Applied Biosystems). Amplifications were performed starting with a 2 min activation step for Amperase UNG at 50°C, 10 min template denaturation step at 95°C, followed by 40 cycles at 95°C for 15s and 60°C for 1 min. Pre-developed TaqMan Assay reagent for GAPDH (4310884E) was used as endogenous control. Absence of amplification from non-reverse-transcribed RNA was confirmed to exclude genomic DNA amplification. Differences in gene expression were calculated by standard 2^−ΔΔCt^ method. For the statistical analysis Student's t test was applied.

## Results

### Abacavir inhibits cell growth, affects cell cycle progression and induces senescence in prostate cancer cell lines

**Figure 1:**
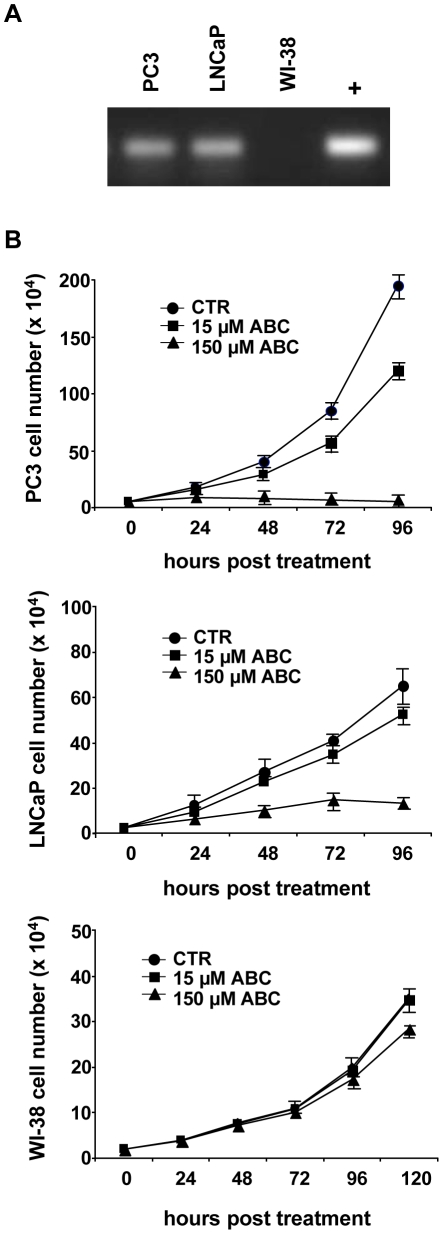
ABC induces a dose-dependent growth inhibition in prostate cancer cell lines. (A) Endogenous RT activity was detected in PC3, LNCaP and WI-38 cells as described in material and methods; (+) positive control reaction with commercial RT. (B) Prostate cancer and normal human fibroblast WI-38 cells were treated with ABC at the concentration of 15 and 150 µM and cultured at the indicated time points. Data shown are representative of at least three independent experiments; bars, ± SD.

First, we evaluated endogenous RT activity in human prostate cancer cells and in non-transformed WI-38 control cells. Cells extracts were used as RT source to reverse transcribe a synthetic RNA. As shown in Figure 1A, RT activity was found in PC3 and LNCaP cells, whereas in WI-38 cells RT activity was at an undetectable level.

To analyze the effect of ABC on cell proliferation rate, cells were grown in the presence of the drug at concentrations of 15 and 150 µM. A dose-dependent growth inhibition was observed in both cell lines (Figure 1B). At 15 µM ABC a considerable reduction in cell growth was revealed after 72 and 96 h of treatment in PC3 and LNCaP respectively. The 150 µM concentration strongly inhibited the growth in both cell lines (Figure 1B). The cytotoxicity of ABC was also examined in non-transformed WI-38 cells. Interestingly, we did not find a significant reduction of cell growth with 15 µM ABC whereas the 150 µM ABC concentration induced only a 20% of cell growth inhibition after 120 h (Figure 1B).

**Figure 2:**
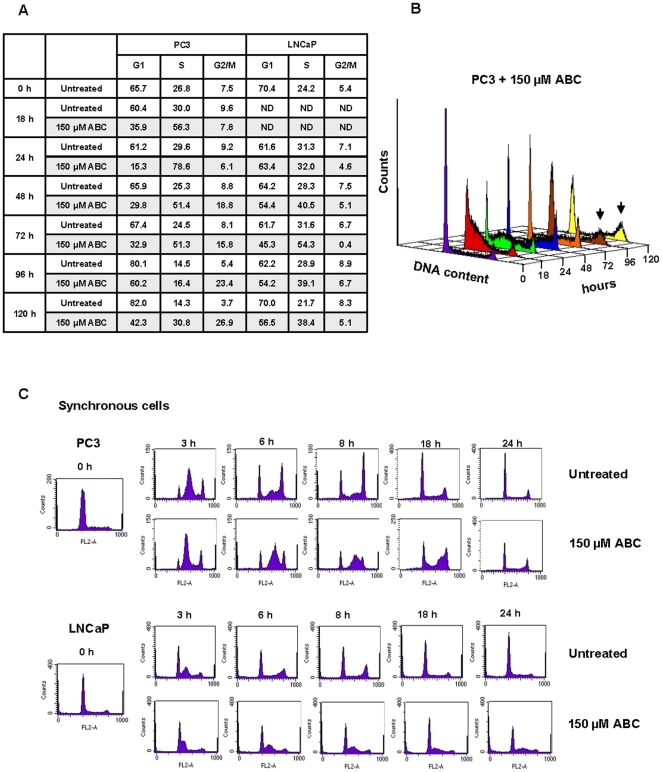
ABC affects cell cycle progression. (A) Cell cycle distribution of PC3 and LNCaP cells exposed to 150 µM ABC. Cells were harvested at the indicated time points, incubated with propidium iodide and DNA content was analysed by flow cytometry. The percentage of cells in each phase is reported. (ND, not done). The data are representative of five independent experiments. (B) A representative experiment of cell cycle progression in PC3 treated cells. The arrows indicate cell accumulation at G2/M phase. (C) PC3 and LNCaP cells were synchronized in early S phase by aphidicolin and cell cycle distribution was analyzed in treated (150 µM ABC) and untreated cells.

To investigate whether ABC could affect the cell cycle progression, prostate cancer cells were treated with 150 µM ABC and analyzed at different time points up to 120 h. In PC3 cells a very high accumulation of cells in S phase was seen after 18 and 24 h of treatment (56.3 and 78.6% respectively). This increase was followed by an augment of G2/M cells which became 23.4–26.9% of total population upon 96 and 120 h of treatment (Figure 2A, B). LNCaP treated cells showed predominantly an S phase accumulation reaching 40.5–54.3% after 48 and 72 h of treatment, but no G2/M phase increment was observed. Rather, S phase accumulation seems to remain constant over time (Figure 2A). To further characterize the S phase alteration, cells were synchronized in early S phase by exposing them to the DNA synthesis inhibitor aphidicolin. After release from the aphidicolin block, cells were treated with 150 µM ABC and analysed for cell cycle distribution at different time points. Figure 2C, shows that 3 h after release both synchronized control cells had moved toward the middle S phase, represented by the central peak of the graph, whereas ABC treated cells showed an evident delayed progression through the S phase. After 18 h, a large amount of PC3 treated cells was still present in late S and G2/M phase, whereas LNCaP, as expected, showed a trend to accumulate in S phase (Figure 2C). Noteworthy, ABC was able to lengthen S phase progression also if added in the middle S phase, 3 h after aphidicolin block release (data not shown). All together these data support the hypothesis that ABC could specifically affect DNA replication.

**Figure 3:**
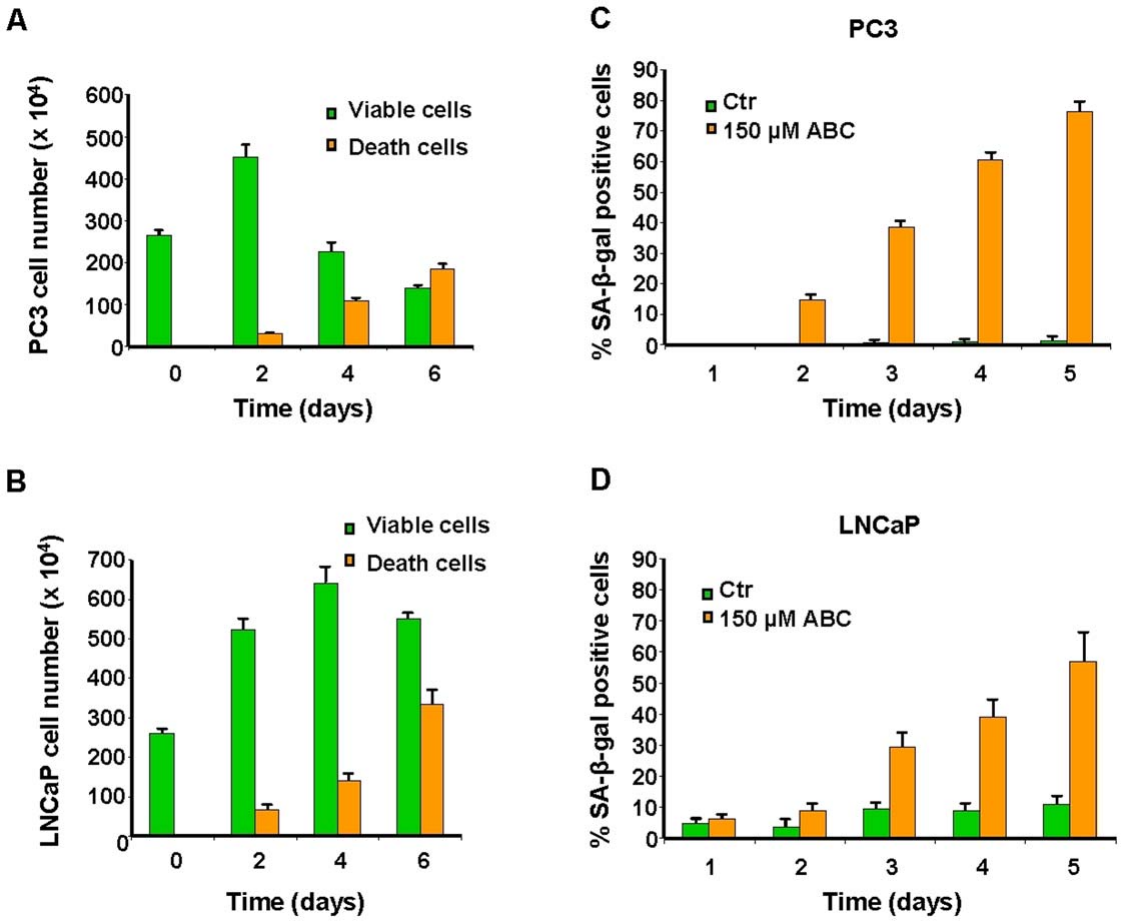
ABC treatment induces senescence and cell death in prostate cancer cells. (A and B) 5×10^4^ cells were seeded in 12-well plates with 150 µM ABC. After 0, 2, 4 and 6 days adherent and floating cells were harvested and resuspended in culture medium containing 0.2% trypan blue for the count of viable and death cells. (C and D) ß-galactosidase activity was evaluated in PC3 and LNCaP cells treated with 150 µM ABC and analyzed at different time points.

Together with proliferation inhibition, a considerable increment of cell death over a period of 6 days was observed with 150 µM ABC (Figure 3A, B). To evaluate whether these phenomena were associated with apoptosis induction or senescence, prostate cancer cells were treated with 150 µM ABC for 5 days and assessed every 24 h for annexin externalisation, nuclear condensation/fragmentation and ß-galactosidase activity at pH 6. No significant increase of annexin positive cells or apoptotic nuclei was observed in treated samples at any time point (data not shown) whereas senescence associated ß-galactosidase activity was significantly induced by ABC and increased with time, reaching about 80% and 50% of positive cells upon 5 days of treatment in PC3 and LNCaP respectively (Figure 3C, D). Moreover, the comparison between cellular senescence and cell death kinetics suggests that the two phenomena are strongly associated. On the contrary, we did not observe cell death nor differences in senescence level in WI-38 control cells treated with 150 µM ABC, compared to untreated cells (data not shown).

### Morphological Changes of PC3 Treated Cells

**Figure 4:**
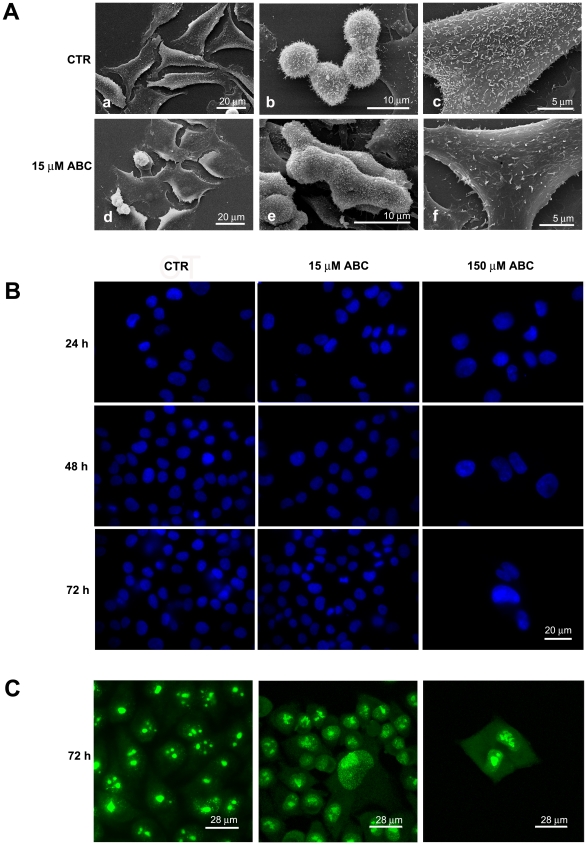
Treatment of PC3 cells with ABC induces distinct morphological changes. (A) SEM image illustrate modification occurring in PC3 cells treated with 15 µM ABC at 48 h. (B) Morphological changes and morphometric analysis of the nuclei stained with Hoechst. Doses and time-points are indicated. (C) Immunofluorescence of nucleoli stained with anti-nucleus human serum.

PC3 cells were further analyzed at morphological level. According to cell cycle alteration and senescence induction, several cell morphological changes were observed after exposure to the drug at both doses. At scanning electron microscopy PC3 cells treated with 15 µM ABC already displayed a more flattened shape (Figure 4A a, d) and a hindrance in division process after 48 h exposure (Figure 4A b, e). In addition, treated PC3 cells show the loss of surface microvilli that suggest an effect of the drug on cytoskeleton organization (Figure 4A c, f). At nuclear level, morphological analysis emphasizes the presence of bilobate and enlarged nuclei, with an increase of the nuclear area becoming evident at 48 h of incubation with 15 µM ABC and 24 h after treatment with 150 µM ABC (Figure 4B). Morphometric and statistical analysis indicated that nuclear areas approximately doubled in 48 h with 150 µM ABC (Table S2). At nucleolar level significant structure modifications were found in the cells exposed for 72 h to both ABC doses. Furthermore, PC3 treated cells nucleoli appear less compact and in some case scattered respect to the control (Figure 4C).

### Abacavir Inhibits Migration and Invasion

**Figure 5:**
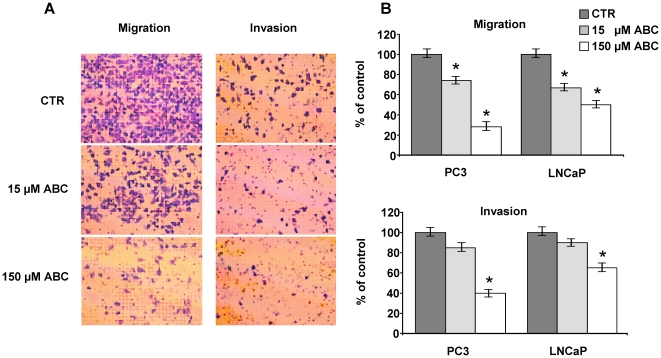
ABC reduces migration and invasion potential of prostate cancer cells. PC3 and LNCaP cells were seeded on the membrane in presence or absence of Matrigel and incubated with 15 and 150 µM ABC for 18 h. (A) representative image of migrating and invading PC3 cells stained with crystal violet. (B) Percentage of migration and invasion decrease respect to untreated cells analysed by Optilab software. (*) corresponds to p<0.001, calculated by the Mann-Whitney test.

Since PC3 and LNCaP cells are of metastatic origin and possess a migratory and invasiveness potential, we investigated the effects of ABC on the motility and invasion processes. Cells were seeded in transwell chambers in the presence or absence of 15 or 150 µM ABC, and allowed to migrate for 18 h at 37°C. A dose-dependent decrease of the area occupied by migrating and invading cells was observed after ABC treatment (Figure 5A, B). Cell migration was significantly reduced in prostate cancer cells at 15 and 150 µM ABC doses (p<0.001). Matrigel cell invasion was significantly inhibited only at the higher ABC dose (Figure 5B).

### Gene Expression Modification Induced by Abacavir in PC3 treated cells

In attempt to find a gene expression signature underlying the morphological and functional changes observed in ABC treated PC3 cells, four biological replicates of both treated and control cells were analyzed on an Illumina Microarray platform 48 h after treatment.

**Figure 6:**
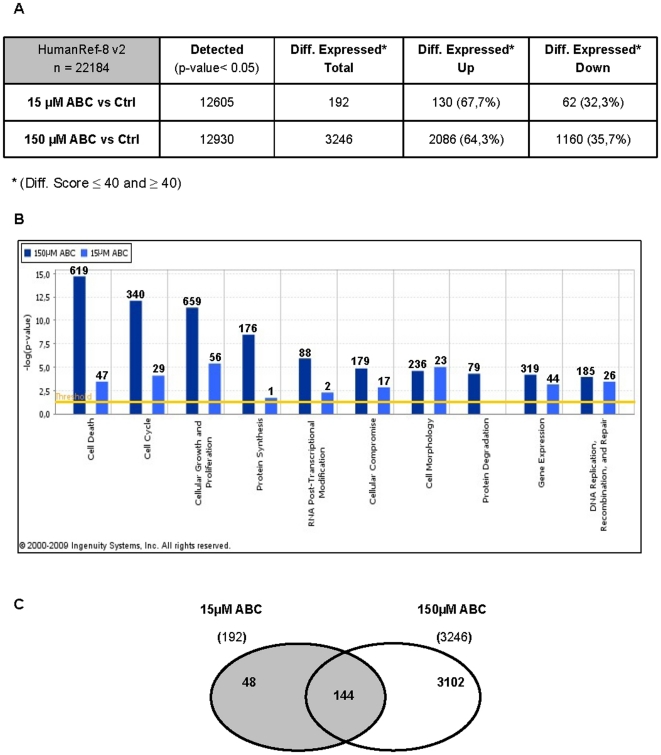
PC3 microarray analysis results. (A) Summarizing chart of the expressed and differentially expressed gene numbers resulting from microarray assay (four biological replicates for each condition). (B) Comparison of the top-ten biological functions at 15 and 150 µM ABC obtained by IPA analysis. On the y-axis the statistical significance, expressed as -log/p-value, is reported. Threshold p-value  = 0.05 (yellow line). The number of the genes involved in each function is reported on the top of each bar. (C) Venn diagram showing the fraction of common genes differentially expressed in PC3 cells at 15 and 150 µM ABC.

Analysis of the microarray data showed that 192 genes out of 12605 detected and 3246 out of 12930, were differentially expressed (p<0.0001) at 15 and 150 µM ABC concentrations respectively, most of which resulted up-regulated (Figure 6A and Table S3).

A function analysis of the differentially expressed genes was performed using Ingenuity Pathways Analysis (IPA) system (Figure 6B). Interestingly, the comparative analysis of the top-ten biological functions shows that the major cellular functions affected by the treatment were the same for the two concentrations, indicating a specific and dose-dependent effect of the drug. The functions described in Figure 6B are consistent and representative of both morphological and functional changes observed at phenotypical level: recovery of cell death pathways, alterations of cell cycle and proliferation rate, changes of cellular morphology. Other significantly affected functions were “RNA Post-Transcriptional Modifications”, “Gene Expression” and “DNA Replication, Recombination and Repair”. Genes clustered in the different functions are listed in Table S4.

The data sets obtained at the two doses were successively compared in a Venn diagram and 144 genes resulted to be shared between the two lists (Figure 6C, Table S5). Among them, the most represented Gene Ontology (GO) terms were identified using the DAVID “Functional Annotation Chart” tool including the three categories: Biological Processes, Cellular Components and Molecular Functions. Functional annotation clusters were identified for each category, with enrichment scores ranging from 1.33 to 8.63 (Table 1). Interestingly, the GO “Cellular Component” category identified 20 genes all belonging to the nuclear compartments and specifically to nuclear part, chromatin remodelling complex and chromosome terms (Table 2).

**Table 1:** DAVID Functional Annotation analysis of the 144 differentially expressed genes.

Category	Term	Genes number	%	pValue	Fold enrichment
Biological Process	GO:0006979∼response to oxidative stress	5	3.57	1.07E-02	5.75
	GO:0043412∼biopolymer modification	26	18.57	1.91E-02	1.56
	GO:0006464∼protein modification process	25	17.86	2.10E-02	1.57
	GO:0043687∼post-translational protein modification	22	15.71	2.21E-02	1.64
	GO:0007275∼multicellular organismal development	26	18.57	3.88E-02	1.47
	GO:0006333∼chromatin assembly or disassembly	5	3.57	3.91E-02	3.87
	GO:0032502∼developmental process	35	25.00	4.76E-02	1.33
Cellular Component	GO:0044428∼nuclear part	18	12.86	2.59E-02	1.73
	GO:0016585∼chromatin remodeling complex	3	2.14	4.61E-02	8.63
	GO:0005694∼chromosome	8	5.71	4.72E-02	2.40
Molecular Function	GO:0008047∼enzyme activator activity	8	5.71	1.53E-02	3.05
	GO:0051082∼unfolded protein binding	5	3.57	2.72E-02	4.35
	GO:0005524∼ATP binding	20	14.29	3.00E-02	1.64
	GO:0032559∼adenyl ribonucleotide binding	20	14.29	3.34E-02	1.62
	GO:0004674∼protein serine/threonine kinase activity	9	6.43	3.52E-02	2.36
	GO:0016773∼phosphotransferase activity, OH as acceptor	12	8.57	4.71E-02	1.91
	GO:0016209∼antioxidant activity	3	2.14	4.88E-02	8.36

**Table 2:** List of the genes clustered into the “Cellular Component” ontology term.

GENE_SYMBOL	GENE NAME
AHCTF1	AT HOOK CONTAINING TRANSCRIPTION FACTOR 1
ANAPC5	ANAPHASE PROMOTING COMPLEX SUBUNIT 5
BANF1	BARRIER TO AUTOINTEGRATION FACTOR 1
CBX6	CHROMOBOX HOMOLOG 6
CLASP1	CYTOPLASMIC LINKER ASSOCIATED PROTEIN 1
DNAJB9	DNAJ (HSP40) HOMOLOG, SUBFAMILY B, MEMBER 9
GEMIN4	DKFZP434B131 PROTEIN
HNRPA3	HETEROGENEOUS NUCLEAR RIBONUCLEOPROTEIN A3
LEMD1	LEM DOMAIN CONTAINING 1
MCM2	MCM2 MINICHROMOSOME MANTEINANCE DEFICIENT 2, MITOTIN (S.CEREVISIAE)
NAP1L4	NUCLEOSOME ASSEMBLY PROTEIN 1-LIKE 4
NSUN2	NOL1/NOL2/SUN DOMAIN FAMILY, MEMBER 2
PCAF	P300/CBP-ASSOCIATED FACTOR
POLA2	POLYMERASE (DNA DIRECTED), ALPHA 2 (70KD SUBUNIT)
SMARCC1	SWI/SNF RELATED, MATRIX ASSOCIATED, ACTIN DEPENDENT REGULATOR OF CHROMATIN, SUBFAMILY C, MEMBER 1
SMARCD3	SWI/SNF RELATED, MATRIX ASSOCIATED, ACTIN DEPENDENT REGULATOR OF CHROMATIN, SUBFAMILY D, MEMBER 3
SNAPC4	SMALL NUCLEAR RNA ACTIVATING COMPLEX, POLYPEPTIDE 4, 190KDA
SNRPB2	SMALL NUCLEAR RIBONUCLEOPROTEIN POLYPEPTIDE B2
TOR1A	TORSIN FAMILY 1, MEMBER A (TORSIN A)
YY1	YY1 TRANSCRIPTION FACTOR

### Abacavir Modulates LINE-1 mRNA Expression

**Figure 7:**
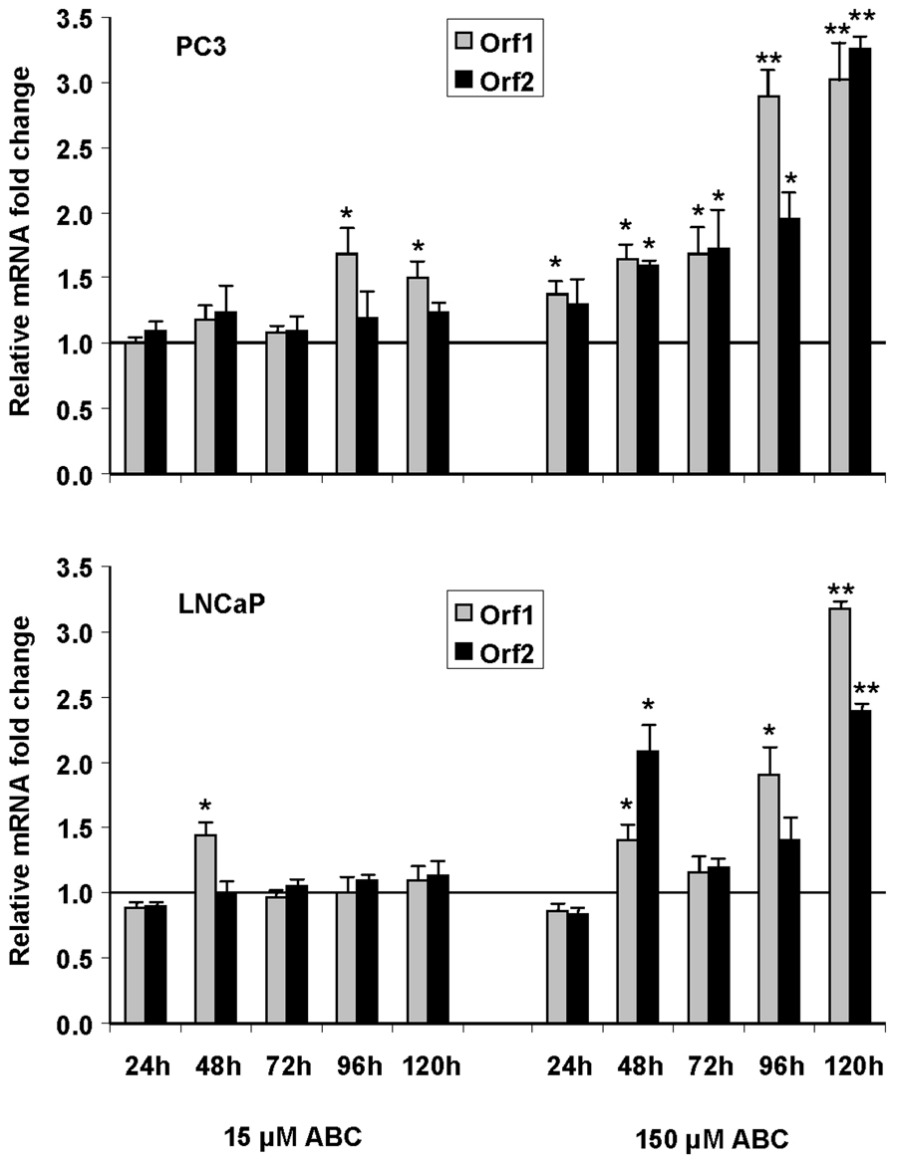
Impact of ABC treatment on LINE-1 mRNA expression. Relative levels of LINE-1 mRNA transcripts (ORF1 and ORF2) in PC3 and LNCaP cells treated with 15 and 150 µM ABC at different time points. Data are expressed as mean ± SD. The (*) and (**) symbols denote a significant difference compared to untreated cells, with a p value <0.05 and <0.01 respectively.

Due to the evidence supporting a relationship between RT activity, LINE-1 expression and cancer cell reprogramming [18]–[21], we decided to investigate the ABC effect on the expression of this class of retrotransposons. Specifically, LINE-1 consist of a 5′ UTR (untranslated region), two open reading frames (ORF1 and ORF2) and a 3′ UTR terminating in a poly (A) tail. ORF1 encodes a protein with RNA binding capacity while ORF2 encodes a protein with endonuclease and reverse transcriptase functions [7]. The cDNAs obtained from prostate cancer cells, untreated and treated with the two ABC doses and harvested at 24, 48, 72, 96 and 120 h, were amplified by Real-Time PCR with primers and probes that recognize conserved regions of LINE-1 ORF1 and ORF2 coding sequences. As shown in Figure 7, a dose and time-dependent increment of mRNAs expression level was observed either in PC3 and LNCaP cells for both ORF1 and ORF2 transcripts, indicating that LINE-1 could play a role in the molecular and functional changes observed upon ABC treatment.

## Discussion

A large number of evidence has confirmed the association between high level of endogenous RT expression and transformed/tumoral cell phenotype [11], [17]. Previous studies with allosteric RT inhibitors, NNRTIs, suggest that LINE-1-encoded RT may be regarded as a novel molecular target in cancer therapy.

In this work we show the antitumor activity of Abacavir, a nucleoside reverse transcription inhibitor (NRTI) on PC3 and LNCaP human prostate cell lines of metastatic origin. Furthermore, we report for the first time that ABC treatment can modulate the LINE-1 mRNA expression.

ABC significantly reduced cell growth, inducing a delay in cell cycle S phase progression in prostate cancer cells. This slow down leads to a consequent arrest in G2/M phase in PC3 cells while LNCaP cells accumulates in S phase. The effect on cell cycle became evident few hours after treatment and cells progressively enter a state of senescence. The appearance of senescence-associated morphological changes and SA-β-gal expression increased gradually in PC3 and LNCaP cells reaching 80% and 50% respectively in 5 days with resulting cell death.

Contrasting tumor cell migration and invasion is a central issue in prostate cancer. It is well known that the presence of metastasis decreases patient's likelihood of survival and that the treatment options currently available are rarely able to cure metastatic forms. Here we report that together with the effect on cell cycle, ABC impairs invasion and migration potential of PC3 and LNCaP cells.

Based on the gene expression profiles of PC3 cells, we provide preliminary insights into the relationship between ABC treatment and gene patterns involved in specific biological functions. The IPA analysis of dose-response data generated super imposable functional clustering at the two ABC concentrations, highlighting a direct correlation between the gene expression pattern and the morphological and functional modifications observed. Further analysis of the 144 differentially expressed genes common to the two ABC doses, drew attention on the GO-Terms related to the nuclear compartment and to those genes involved in “chromatin remodeling complex”, which emerge with a high enrichment score. It can be speculated that ABC could affect the chromatin status, slowing down S phase and exposing the cells to numerous endogenous and/or exogenous replication stresses. This condition, where ABC selectively renders tumor cells more vulnerable to DNA-damaging agents, may be relevant in strategies that combine ABC treatment with other conventional cancer therapies.

Similar effects on cell cycle alteration and senescence induction were previously reported by Rossi et al. [30] in medulloblastoma ABC treated cells. The authors observed a cell growth inhibition with the appearance of senescence features after one week of 350–750 µM ABC treatment, associating these phenomena to the down-regulation of telomerase activity. Nevertheless, it must be noted that in our experimental model we observed a prompt slowdown of S phase and an early appearance of senescence phenotype with 150 µM ABC. Since senescence elicited by inhibitors of the telomerase-associated RT usually requires a higher number of replication events [31], we suggest that the senescence induced by ABC can be dependent on the effect exerted on endogenous RT of non-telomeric origin.

The most characterized and abundant retrotransposons in the mammalian genome are LINE-1 sequences. About 79% of mammalian genes contain at least one LINE-1 segment in their transcription unit, mainly within intronic regions, with the poorly expressed genes containing a larger amount of LINE-1 sequences respect to the highly expressed genes [32].

Although expression of LINE-1 is barely detected in most normal somatic cells due to epigenetic suppression, their expression is elevated in many cancer cells [33], [34]. A large body of data show that LINE-1 retrotransposition can be increased by a number of environmental stress signals, such as benzo(a)pyrene, UV and γ irradiation, heat shock, cyclohexamide, viral infection, heavy metals, indicating that these elements may play an important role in cancer etiology [35]–[40]. Moreover, despite the great majority of LINE-1 are retrotranspositionally inactive due to 5′ truncations, active transcription and translation of these elements have been recently detected in a variety of cell types and implicated as a potential regulator for cellular processes [41]–[43].

Here we show that in prostate cancer cells ABC treatment induces a dose- and time-dependent increase of LINE-1 ORF1 and ORF2 transcripts. At present we cannot determine how the inhibition of the LINE-1 cDNA elongation could cause an increase of the ORF1 and ORF2 transcripts, nor if the increase is due to a bona fide transcription induction or to a stabilization of LINE-1 mRNAs. Further investigations are required to elucidate the molecular mechanism underlying this phenomenon.

Nevertheless, our results are consistent with previous studies demonstrating how in cancer cells an increase in LINE-1 expression, obtained by synthetic constructs, has a genome-destabilizing effect, inducing impairment in cell cycle progression that leads to a senescence-like state [44], [45]. In addition, even if active cell divisions are required for LINE-1 retrotransposition [46], a number of evidence suggests that this event itself is not needed to cause “toxicity”. In fact, the overexpression of an ORF2 construct alone is able to reduce the number of viable cells [47]. Interestingly, a recent study by Belancio et al. [48] shows that in normal human fibroblasts immortalized with hTERT, exogenous induction of LINE-1 expression (full-length or spliced ORF2 mRNA) leads to a senescence-like phenotype as a consequence of DNA damage. Beside its role of reverse transcriptase, ORF2 also encodes for the endonuclease activity known to induce DNA double strand breaks [44], [48].

Thus, if endogenous LINE-1 expression induces a wide modification in gene expression independently of retrotransposition, this implies a more important role for LINE-1 products (mRNAs, cDNAs, proteins). Actually, little is known about the genetic interaction of LINE-1 with other genes within the mammalian genome or the complex molecular mechanisms regulating LINE-1 activity. *In silico* studies of the human genome have shown that LINE-1 may coordinate gene transcription by providing regulatory sequences that control gene expression or silencing. [43], [49]–[51].

In conclusion, we propose ABC as an anticancer agent able to selectively affect multiple cellular functions and trigger senescence in PC3 and LNCaP cells. We also suggest that ABC-induced senescence may be related to the up-regulation of LINE-1 transcripts in prostate cancer cells. Our findings strongly support the emerging concept of endogenous RT as an attractive target in cancer therapy, providing an innovative strategy to circumvent the difficulties related to the genetic heterogeneity of cancer phenotypes. It would be interesting to evaluate in preclinical studies the efficacy of ABC in combination with docetaxel, a chemotherapeutic agent used in hormone-refractory metastatic prostate cancer. Since Abacavir is clinically approved for human use in AIDS therapy, drug repositioning in prostate and, possibly, in other cancer forms resistant to current therapies, could accelerate the traditional drug development.

## Supporting Information

Table S1 Primers and probes sequences for LINE-1 mRNA.
(0.03 MB DOC)

Table S2 Morphometric analysis results.
(0.02 MB DOC)

Table S3 List of differentially expressed genes at 15 and 150 µM ABC in PC3 cells.
(1.73 MB XLS)

Table S4 Lists of differentially expressed genes clustered in each IPA function.
(0.21 MB XLS)

Table S5 List of the common differentially expressed genes at 15 and 150 µM ABC in PC3 cells.
(0.08 MB XLS)
