# Supplementary material for: The Reverse Transcription Inhibitor Abacavir Shows Anticancer Activity in Prostate Cancer Cell Lines
Source: PLoS One. 2010 Dec 3;5(12):e14221. doi: 10.1371/journal.pone.0014221 (PMC2997057; doi:10.1371/journal.pone.0014221)
Supplement: Table S1 — Primers and probes sequences for LINE-1 mRNA. (0.03 MB DOC) [file pone.0014221.s001.doc]

Table S1. Primers and probes sequences for LINE-1 mRNA

_______________________________________________________________________

Target Sequence 5’ to 3’

_______________________________________________________________________

ORF1 Forward TTGGAAAACACTCTGCAGGATATTAT

Reverse TTGGCCTGCCTTGCTAGATT

Probe FAM-CAGGAGAACTTCCC-MGM

ORF2 Forward AAAATACTGGCAAACCGAATCC

Reverse TGAAGCCCACTTGATCATGGT

Probe FAM-AGCACATCAAAAAGCTTAT-MGM

______________________________________________________________________

The primers and TaqMan minor groove binding probes (MGM) were designed with Primer Express software, version 2.0 (Applied Biosystems, Foster City, Calif.), based on a consensus sequence obtained by aligning all the human full-length intact LINE-1 sequences from the L1base ([http://l1base.molgen.mpg.de](http://l1base.molgen.mpg.de/)) using the BioEdit Sequence Alignment Editor, version 7.0.9.

The human L1 database includes the intact in the two ORFs, full length L1s (FLI-L1s, 145 entries) and L1s with intact ORF2 but disrupted ORF1 (ORF2-L1s, 103 entries). Moreover, due to their regulatory potential, the full length (>6000bp) non-intact L1s (FLnI-L1s, 11653 entries) are also included in the database.

Blast analysis performed with the two L1 probe sequences used in this work, indicates that the number of HSP's (High-scoring Segment Pair) better than 10.0 without gapping are the sequent:

ORF1 probe: 143 / 145 in FLI-L1s

85 / 103 in ORF2-L1s

6487 / 11653 in FLnI-L1s

ORF2 probe: 144 / 145 in FLI-L1s

99 / 103 in ORF2-L1s

8111 / 11653 in FLnI-L1s
