# Supplementary material for: The Reverse Transcription Inhibitor Abacavir Shows Anticancer Activity in Prostate Cancer Cell Lines
Source: PLoS One. 2010 Dec 3;5(12):e14221. doi: 10.1371/journal.pone.0014221 (PMC2997057; doi:10.1371/journal.pone.0014221)
Supplement: Table S2 — Morphometric analysis results. (0.02 MB DOC) [file pone.0014221.s002.doc]

Table S2. Morphometric analysis results

CTR 15 µM p-value 150 µM p-value

________________________________________________________________________________

Nuclei

24 h 191.07 ± 78.01 180.46 ± 72.01 ns* 256.46 ± 91.41 <0.001

48 h 162.91 ± 83.20 206.56 ± 95.99 <0.001 325.80 ± 127.38 <0.001

72 h 143.16 ± 80.46 138.12 ± 76.15 ns 363.34 ± 118.75 <0.001

Nucleoli

72 h 22.31 ± 13.90 29.63 ± 18.89 <0.001 129.95 ± 26.26 <0.001

________________________________________________________________________________

* not significant
